# Sign learning of hearing children in inclusive day care centers—does iconicity matter?

**DOI:** 10.3389/fpsyg.2023.1196114

**Published:** 2023-08-16

**Authors:** Madlen Goppelt-Kunkel, Anna-Lena Stroh, Barbara Hänel-Faulhaber

**Affiliations:** ^1^Department of Special Education, Faculty of Education, Universität Hamburg, Hamburg, Germany; ^2^Faculty of Psychology, Institute of Psychology, Jagiellonian University, Kraków, Poland

**Keywords:** iconicity, sign language, word learning, special educational needs, disabilities, day care centers, inclusion

## Abstract

An increasing number of experimental studies suggest that signs and gestures can scaffold vocabulary learning for children with and without special educational needs and/or disabilities (SEND). However, little research has been done on the extent to which iconicity plays a role in sign learning, particularly in inclusive day care centers. This current study investigated the role of iconicity in the sign learning of 145 hearing children (2;1 to 6;3 years) from inclusive day care centers with educators who started using sign-supported speech after a training module. Children’s sign use was assessed via a questionnaire completed by their educators. We found that older children were more likely to learn signs with a higher degree of iconicity, whereas the learning of signs by younger children was less affected by iconicity. Children with SEND did not benefit more from iconicity than children without SEND. These results suggest that whether iconicity plays a role in sign learning depends on the age of the children.

## Introduction

The role of iconicity has gained increasing interest in word-learning processes (for reviews, see Dingemanse et al., 2015; Nielsen and Dingemanse, 2021). Sign languages are produced and received in the visual–spatial modality. For this reason, they can be used to express iconic aspects of concepts more directly than spoken languages by representing a physical object’s shape or handling via hand forms or hand movements (Perniss et al., 2010; Perlman et al., 2018). Therefore, sign language studies provide a promising tool for directly assessing the influence of iconicity on word learning (for a review, see Perniss and Vigliocco, 2014).

A range of experimental studies has demonstrated that signs and gestures can support word learning for children with and without SEND (e.g., Kay-Raining Bird et al., 2000; Nunes, 2008; Lüke and Ritterfeld, 2014). However, there is little research on the role of iconicity in this process in heterogenous groups of children. Therefore, the present study investigated whether iconicity affected sign learning in a large sample of hearing non-signing preschool children in inclusive day care centers. We examined the extent to which characteristics such as age or SEND play a role in this.

### Sensitivity to iconicity

Iconicity is the term used when aspects of linguistic or other communicative forms resemble aspects of the objects, activities or other concepts to which they refer (Perniss and Vigliocco, 2014; Dingemanse et al., 2015). This applies to spoken and signed languages, as well as gestures (Perniss and Vigliocco, 2014). As iconicity and arbitrariness coexist within languages (Lockwood and Dingemanse, 2015), words and signs are not either exclusively iconic or arbitrary, but rather differ in their degree of iconicity.

There is now mounting evidence that iconicity has an effect on lexical learning in different languages and modalities (for reviews on spoken languages, see Lockwood and Dingemanse, 2015, Nielsen and Dingemanse, 2021; for a review on sign languages, see Ortega, 2017). In spoken languages, iconicity includes onomatopoeia, i.e., words representing sounds of animals, vehicles, or other objects, as well as sound symbolism, i.e., phonemes or words that match properties of their referents such as their shape or movement (Ota et al., 2018). Onomatopoeic words are more prevalent in the vocabulary of young children as well as in child-directed speech and are discussed to facilitate vocabulary acquisition (Ota et al., 2018; Laing, 2019; Motamedi et al., 2021). Accordingly, sound symbolism is already perceived and exploited by babies (Peña et al., 2011; Ozturk et al., 2013) and continues to be involved in vocabulary learning by children of different ages (Maurer et al., 2006; Imai et al., 2008; Massaro and Perlman, 2017; Tzeng et al., 2017). In addition, early acquired words are more likely to be iconic than later acquired words (Perry et al., 2015, 2017; Massaro and Perlman, 2017; Perlman et al., 2017; Sidhu et al., 2021).

Regarding the role of iconicity in sign learning, the results from previous studies are more inconsistent. In sign language, iconicity represents the visual resemblance between aspects of a sign form and its meaning (Nielsen and Dingemanse, 2021). One of the first longitudinal studies on signing babies of deaf parents (aged 0;10 at the beginning to 1;6 years at the end) found that the babies’ sign lexicon did not consist predominantly of iconic signs (Orlansky and Bonvillian, 1984). By contrast, recent studies with larger sample sizes of native-signing deaf children (mostly aged less than 3 years) have found that the more iconic the signs were, the more likely they were produced (Caselli and Pyers, 2017 for American Sign Language; Novogrodsky and Meir, 2020 for Israeli Sign Language; Sümer et al., 2017 for Turkish Sign Language with a sample of deaf and hearing native signing children; and Thompson et al., 2012 for British Sign Language) and comprehended (Thompson et al., 2012 for British Sign Language).

Diverging results have also been reported for the interaction between iconicity and age. An analysis of the active and passive vocabulary of native-signing deaf children using British Sign Language revealed that older children (1;9 to 2;6 years) benefit more from iconicity than younger children (0;11 to 1;8 years; Thompson et al., 2012), while another study with a larger sample of native-signing deaf children (0;8 to 2;11 years) measuring sign production in American Sign Language could not replicate this age effect, maybe due to a ceiling effect (Caselli and Pyers, 2017). A study on deaf children using Israeli Sign Language with a wider age band (0;8 to 7;2 years) again observed a different pattern: While the youngest children (0,8 to 2;0 years) only produced signs with the highest degree of iconicity likely, slightly older children (2,1 to 4;6 years) overall produced signs with a higher degree of iconicity more likely than signs with a lower degree of iconicity (Novogrodsky and Meir, 2020). In children older than 4;7 years there was also a ceiling effect making it impossible to draw conclucions on the role of iconicity in this age group (Novogrodsky and Meir, 2020). But an experimental study on deaf children with iconic gestures found that the 3-year-olds (Mean age: 3.67 years) benefited to a similar degree from iconicity as the 4- to 5-year-olds (Mean age: 5.04 years) in spontaneously mapping and learning iconic gestures (Magid and Pyers, 2017).

Experimental studies with hearing children suggest that hearing children do not benefit significantly from iconicity in signs or gestures until they are somewhat older: Magid and Pyers (2017) found that 4- to 5-year-olds spontaneously mapped iconic gestures to referents above chance whereas hearing 3-year-olds did not. Moreover, the hearing 4- to 5-year-olds relied significantly more on iconicity in learning novel gestures than the hearing 3-year-olds (for similar results see Marentette and Nicoladis, 2011). Based on these observations, Magid and Pyers (2017) suggest that acquiring a sign language, which is rich in iconicity might shift the timepoint when children capitalize on iconicity to an earlier age. This dovetails nicely with the results observed by Tolar et al. (2008) who tested the comprehension of iconic signs in non-signing children. They observed that the 2.5-year-olds mainly guessed, the 3-year-olds predominantly matched iconic signs and their referents correctly, 3.5- and 4-year-olds recognized the meanings more reliably, and the 4.5- to 5-year-olds reached almost adult levels of accuracy. This supports the idea that cognitive development and growing world knowledge allow the children to exploit iconicity with increasing age more effectively for lexical learning (Meier et al., 2008; Marentette and Nicoladis, 2011; Perniss and Vigliocco, 2014; Magid and Pyers, 2017; Ortega, 2017).

Accordingly, in most studies on hearing adult signing beginners, iconicity aids production and recognition of signs in the early stages of learning (e.g., Lieberth and Gamble, 1991; Baus et al., 2013; Ortega et al., 2022; for an overview, see Chen Pichler and Koulidobrova, 2016). Moreover, iconicity seems to speed lexical access in L2 sign language learners (Mott et al., 2020) and it has been shown to facilitate lexical access in deaf native signers when signs and their corresponding pictures overlap (McGarry et al., 2023). It has been suggested that iconic gestures serve as manual cognates for sign-naïve adults at first exposure to iconic signs (Ortega et al., 2020, 2022). Nevertheless, iconic signs with high and low overlap to iconic gestures have been shown to be learned equally successfully by hearing non-signers (Ortega et al., 2020). Hearing adult sign language learners may generelly rely on their experiences with iconic gestures when learning a language in a different modality (Kurz et al., 2023).

### The role of iconicity of gestures and signs in word learning

Gestures and signs have been shown to support word learning in typically developing children (e.g., Ellis Weismer and Hesketh, 1993; Goodwyn et al., 2000; Capone Singleton, 2012; Mumford and Kita, 2014; Vogt and Kauschke, 2017; for second language learning, see Tellier, 2008) and in hearing children with SEND (for an overview, see Toth, 2009), such as specific language impairments (Ellis Weismer and Hesketh, 1993; Vogt and Kauschke, 2017), Down syndrome (e.g., Kay-Raining Bird et al., 2000), or children with autism spectrum disorder who exhibit severely delayed or absent language development (Bonvillian et al., 1981; Goldstein, 2002; Nunes, 2008). This includes both beneficial effects on the learning of spoken words via simultaneously offered signs or gestures and the acquisition of a functional vocabulary with signs when spoken language cannot be acquired or cannot yet be acquired sufficiently (see Luftig, 1982).

One reason why signs or gestures may facilitate word learning is iconicity (e.g., Bonvillian et al., 1981; Lloyd et al., 1985; Kay-Raining Bird et al., 2000; Nunes, 2008; Capone Singleton, 2012; Lüke and Ritterfeld, 2014). It has been speculated that iconicity supports semantic representation (Capone Singleton, 2012; Lüke and Ritterfeld, 2014) and requires less symbolic processing (Bonvillian et al., 1981; Mirenda and Erickson, 2000).

In typically developing children, data by Lüke and Ritterfeld (2014) and Vogt and Kauschke (2017) suggest that iconic gestures may be more effective than arbitrary gestures to support novel word learning. Numerically there seems to be an advantage to recognizing words presented with iconic gestures in contrast to arbitrary ones. However, statistical comparisons were not significant. The two studies had small sample sizes and possibly ceiling effects, thus lacking power (Lüke and Ritterfeld, 2014; Vogt and Kauschke, 2017). For signs of a sign language, some older work reports that typically developing preschoolers learn transparent, i.e., guessable signs (Brown, 1977 in Luftig, 1982) and translucent signs, whose meaning is clear when the relationship is known (Page, 1981 in Luftig, 1982) more easily than less transparent or translucent signs.

Regarding hearing children with SEND, several researchers have suggested that iconicity might be one reason why children with certain special educational needs or disabilities learn signs or gestures more easily than spoken words (Bonvillian et al., 1981; Lloyd et al., 1985). In one of the first studies (Konstantareas et al., 1978), five school aged children with autism spectrum disorders were shown to learn significantly more iconic signs than arbitrary signs. Furthermore, iconic signs have been reported to represent a large part of the early vocabulary of children with autism spectrum disorders (Bonvillian et al., 1981). Also, children with a complex intellectual disability (ID) have been shown to benefit from sign iconicity in word learning (Griffith and Robinson, 1980 in Griffith et al., 1981 and Luftig, 1982; Lloyd et al., 1985; for a review, see Luftig, 1983). Accordingly, for adults with ID, translucent signs have been shown to be acquired more effectively and to be more functional than other signs (Meuris et al., 2014). Overall, the signed modality does not seem to present any particular disadvantages for adults with cognitive disabilities when compared to neurotypical learners (Joyce et al., 2023).

Gesture research on children with atypical development also sheds light on how children with different special educational needs or disabilities leverage communication in the visual–spatial modality with respect to iconicity. Hearing children with Down syndrome (DS), aged between 3;0 and 8;3 years, have been observed to exhibit a higher frequency of gesture usage compared to typically developing children (Pirchio et al., 2003; Stefanini et al., 2007). Crucially, a greater proportion of these gestures among children with DS are iconic in nature (Pirchio et al., 2003, Stefanini et al., 2007; for an overview, see Capirci et al., 2010). Concerning hearing children with Williams syndrome (WS), there are contradictory findings: Despite some older studies reporting children with WS to use spontaneous gestures less and later than typically developing children (for an overview, see Capirci et al., 2010), a small study in preschool children (3;3–4;3 years) with WS (Pirchio et al., 2003) observed a use of gestures similar to typically developing children. Moreover, school-aged children with WS (9;5–12;9 years) produced more iconic gestures in a picture naming task than typically developing children suggesting that they may compensate word finding difficulties in spoken language via the visual–spatial modality (Bello et al., 2004). Although gesture research on children with autism spectrum disorder has reported that this group of children uses less spontaneous gestures than typically developing children, recent research in school-aged children with autism spectrum disorder (6;11–11;4 years) reveals that these children could nevertheless benefit from iconic gestures in the recall of spoken language (Dargue et al., 2021). For preschoolers with specific language impairment (SLI), Vogt and Kauschke (2017) observed in an experimental study that they seemed to benefit more from iconic gestures than from deictic ones when learning novel words. To our knowledge, no study has directly compared the natural learning of arbitrary and iconic signs in preschool children with and without SEND.

### The current study

We explored if the degree of iconicity influences the acquisition of signs in a larger sample of hearing non-signing preschool children. Sign learning was assessed with a questionnaire that was filled out by the educators for each child. We also investigated whether age, SEND, or language abilities influence the acquisition of signs. We hypothesized that children would learn more high-iconicity signs than low-iconicity signs. Moreover, we hypothesized that the older children in our sample would learn more iconic signs than the younger ones due to their advanced ability to recognize iconicity. The better benefit from iconicity with increasing age might arise due to growing world knowledge and/or cognitive development enabling children to link iconic aspects of the signs and properties of their referents more successful (Marentette and Nicoladis, 2011; Perniss and Vigliocco, 2014; Magid and Pyers, 2017; Ortega, 2017). Using a larger sample of hearing non-signing children with a fairly wide age range, we wanted to observe the extent to which these effects occur in sign learning in the natural setting of an inclusive day care group. Based on Konstantareas et al. (1978) and Lloyd et al. (1985), we assumed that children with SEND could possibly benefit more from iconicity in sign learning than children without SEND.

## Materials and methods

Through in-service training, educators from 25 day care centers learned signs from German Sign Language (German: *Deutsche Gebärdensprache*, short: DGS). It was decided to offer signs of DGS (rather than gestures) in order to introduce alternative forms of communication into the day care centers. The signs were selected according to the German core and fringe vocabulary (Boenisch and Sachse, 2007) which consists of nouns, verbs, adjectives, and function words enabling communication (Boenisch, 2013). In total, 232 signs that are often used in daily kindergarten routines were chosen.

**Figure 1:**
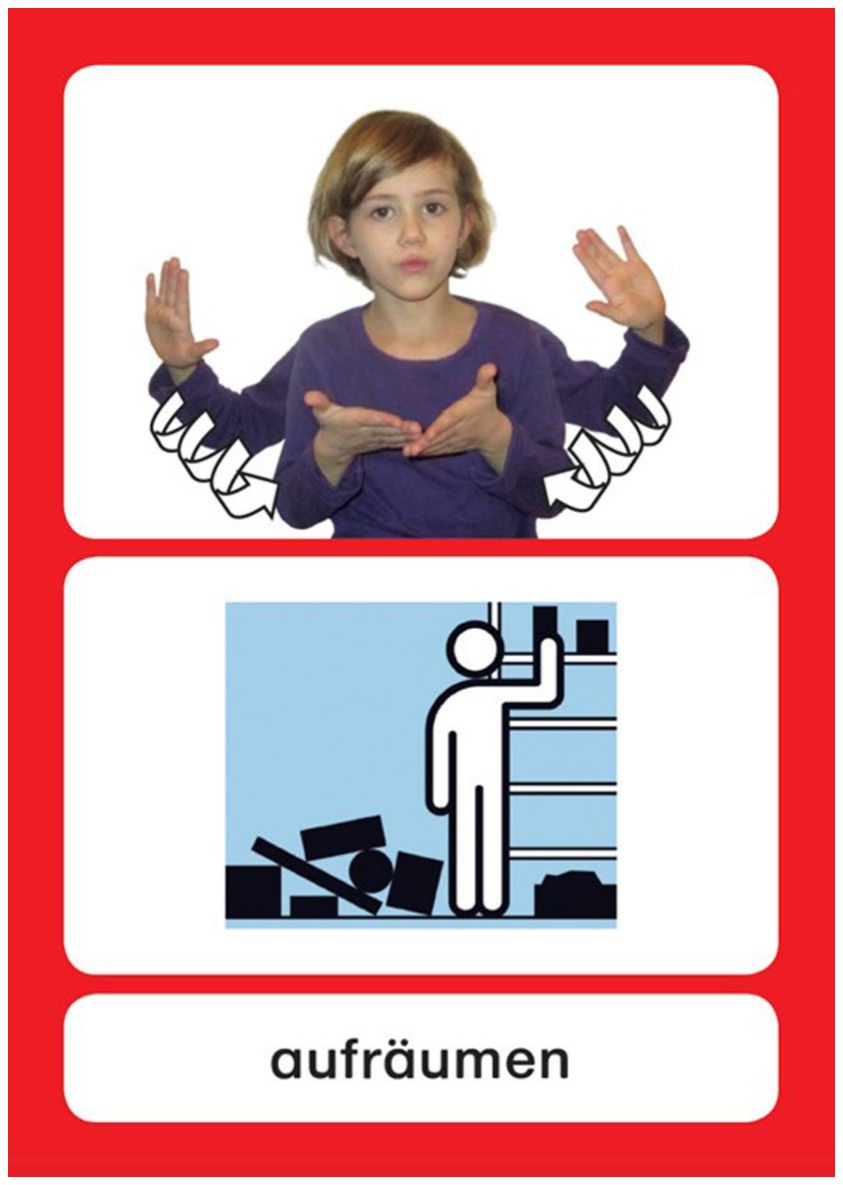
Card for “to tidy up” (German: “aufräumen”) with sign, symbol, and word.

These 232 signs were handed out to every day care group printed on cards, each together with a matching symbol (out of the system “METACOM,” Kitzinger, 2018) and the German translation (Sterner et al., 2021, see Figure 1).

The training consisted of a full-day introductory session and eight training sessions, each lasting 2-3 hours, spread over a period of 6 months. The training sessions aimed to reinforce and expand the participants’ knowledge of the learned signs. Educators were encouraged to accompany their speech with signs right after the first day of training consistently with all the children in all activities, if possible. But since this study was conducted in a natural setting, the extent to which the children were exposed to input with signs varied, i.e., depending on the children’s or the educators’ sick leave or the respective play situations.

### Participants

Inclusive day care groups from a large day care provider were randomly selected to participate in the study. In Germany, most of the children are no longer assigned to day care centers according to their disabilities or special educational needs and inclusive day care centers are supposed to care for children irrespective of their need of support. Therefore, the children in our sample differed greatly in their need of support (see Table 1). But no child of our sample was diagnosed to have a permanent hearing loss. Only groups without previous knowledge of signs or sign language were included in order to avoid biases due to already acquired vocabulary at the beginning of the study. In addition, for the present study, only the day care groups with educators who had stated that signing had become an integral part of their work (*n* = 10) and groups without educator self-assessment but with significantly increased sign vocabulary (*n* = 3) were analyzed (Schüler et al., 2021; Schüler and Hänel-Faulhaber, 2022). Obviously, when educators do not use that many signs, or no signs at all, as observed in a study by Marshall and Hobsbaum (2015), measurement and assessment of effects is limited (see also Schüler et al., 2021). This resulted in a final sample of 13 day care groups. From these groups, complete questionnaire data from 145 children were available.

**Table 1:** Demographic characteristics.

	Age	Sex	Languages
Children without SEND (*n* = 112, percentage: 77.2%)	Mean: 4;2 yearsRange: 2;1–6;3 years	Boys: *n* = 49 (43.8%)Girls: *n* = 63 (56.3%)	Monolingual: *n* = 72 (64.3%)Bilingual: *n* = 40 (35.7%)
	*German acquisition (age):*< 24 months: *n* = 98 (87.5%)≥ 24 months: *n* = 14 (12.5%)
	*Languages besides German:* Turkish (11), Russian (7), Polish (5), French (4), without specification (3), Albanian (2), English (2), Persian (2), Portuguese (2), Serbian (2), Arabic (1), Dari (1), Dutch (1), Greek (1), Kurdish (1), Moroccan (1), Spanish (1), and Thai (1)
	Children with SEND (*n* = 33, percentage: 22.8%)	Mean: 4;8 yearsRange: 2;4–6;2 years	Boys: *n* = 26 (78.8%)Girls: *n* = 7 (21.2%)	Monolingual: *n* = 13 (39.4%)Bilingual: *n* = 20 (60.6%)
	*Therapy/assistance:*Speech therapy: *n* = 27 (81.8%)Occupational therapy: *n* = 13 (39.4%)Curative education^1^: *n* = 6 (18.9%)Physical therapy: *n* = 5 (15.2%)Without specification: *n* = 2 (6.1%)No therapy/assistance: *n* = 2 (6.1%)Psychological care: *n* = 1 (3.0%)*Of these:*Combined treatments: *n* = 16 (48.5%)	*German acquisition (age):*<24 months: *n* = 25 (75.8%)≥ 24 months: *n* = 8 (24.2%)	*Languages besides German:* Turkish (5), Russian (4), Polish (2), Albanian (1), Dari (1), Farsi (1), French (1), Greek (1), Hindu (1), Slovak (1), Twi (1), without specification (1)

^1^In Germany, curative education is provided to children with mental, physical, social–emotional and linguistic impairments, which includes children with global developmental disorders, but also children with specific support needs due to, for example, sensory disabilities.

Children’s language skills were assessed with an integrated questionnaire “*Sprachbeurteilung durch Eltern* (SBE-3-KT)” (language assessment through parents; Suchodoletz et al., 2011), which is normed for children between 32 and 40 months. The SBE-3-KT is an easy-to-use language screening with high validity and reliability. It is based on speech production with a focus on vocabulary and basic morphosyntax. Within the possible achievable score range of 0–172, the children in our sample scored an average of 136.14 points. The median, however, was 168 points because most of the children in our sample exceeded the age norm, resulting in a ceiling effect (see Table 2).

**Table 2:** SBE-3-KT screening scores and results.

SBE-3-KT			*N*	Percentage
*Screening score* (*0–172 achievable points*)	Mean: 136.14 pointsMedian: 168 points			
	*Screening result* (*per age group*)	< 32 months	Negative	1	0.7%
(Positive^*^)			(7^*^)	(4.8%^*^)
32–40 months		Negative	12	8.3%
		Positive	7	4.8%
>40 months		(Negative^*^)	(88^*^)	(60.7%^*^)
		Positive	30	20.7%

Negative results indicate age-appropriate language development, and positive results indicate abnormalities in language development.

* Since the SBE-3-KT is not normed for this age group, this test result cannot be used to determine whether language development is age-appropriate.

### Materials

Six months after the implementation of signs to support communication, educators completed a questionnaire about children’s spoken and sign language development (Schüler et al., 2021, see Supplementary Material A). The questionnaire included questions regarding demographic data such as age and sex, language background (monolingual or bilingual), SEND (therapies and/or disabilities), and a language screening via the slightly modified version of the SBE-3-KT (Suchodoletz et al., 2011). The SBE-3-KT consists of a list of 82 words and a grammar section with 15 sentences. We offered the word list also as a sign list and added 12 items that were part of the sign material to include more signs of the training module. In summary, the vocabulary list contained 40 signs that were part of the training module. The educators were asked to indicate which of these items had already been produced by the child more than once. For the scoring and assessment of spoken language skills, only the 82 words of the original SBE-3-KT were evaluated in order to follow the normed scoring procedure.

For measuring the degree of iconicity in sign acquisition, we evaluated the 40 signs of the questionnaire’s vocabulary list that were part of the training material (see Appendix). These signs included 11 nouns, nine verbs, 11 adjectives, and nine signs of other word classes, such as adverbs, pronouns, or prepositions. To assess the iconicity of the signs, we asked hearing university students to rate the iconicity of the 40 signs on a seven-point Likert scale (1 = not iconic at all; 7 = highly iconic), consistent with Vinson et al. (2008, but with deaf native signers) and Caselli et al. (2017). Only participants without knowledge of sign language were included (see Caselli et al., 2017, for a similar procedure for ASL-LEX) to avoid biases due to prior knowledge (Vinson et al., 2008, for BSL). A total of 147 students (mean age: 28;0 years, range: 19–58 years; 109 female, 36 male, 2 diverse) completed the ratings. All participants had normal or corrected-to-normal vision. Overall, nine signs were rated as non-iconic or barely iconic (median degree of iconicity 1 or 2) and nine signs as highly iconic (median degree of iconicity 6 or 7). The remaining 22 signs were assessed to have a median degree of iconicity ranging from 3 to 5. For five signs from our dataset, norms for iconicity are available (Trettenbrein et al., 2021). The mean and median degree of iconicity in our rating is consistent with these values or differs by at most one point (see Appendix).

To assess whether the signed input provided by the educators was biased regarding iconicity, we analyzed video data of the educators and the children during free play in five of the 13 day care groups.

## Analysis

We used *R* (version 4.0.2—R Core Team, 2020) and *glmmTMB* (version 1.0.2.1—Brooks et al., 2017) to perform a generalized linear mixed-effects analysis with a binomial link function. The dependent variable was sign acquisition, following the children’s questionnaire data (acquired = 1, not acquired = 0). As fixed effects, we entered the median *degree of iconicity* (z-scaled), *age* (z-scaled), *language score* (z-scaled), *early* *versus* *late acquisition of German language*^1^ (age of onset of acquisition before *versus* after the age of 24 months), *SEND* (yes, no) and the interaction terms of these variables with the variable *degree of iconicity*. Fixed effects were classified as significant at *p* < 0.05. To minimize Type II errors, we considered *p* < 0.1 as trends.

*Post hoc* comparisons of significant interactions were conducted using approximate *t*-tests on the estimated marginal means (EMMs) using the *R emmeans* package (version 1.4.8-1, Lenth, 2023). The confidence level of 0.95 was corrected with the Šidák correction for three estimates (Šidák, 1967). The *p*-values were adjusted following the procedure proposed by Tukey (1977).

To ensure that collinearity was not a biasing factor, we checked multicollinearity with the *R performance* package (Version 0.6.1: Lüdecke et al., 2021). For every included variable, there was a variance inflation factor (VIF) under 2.0, which indicates that they were all independent enough from the other variables.

It has been shown that the degree of iconicity of a word or sign can differ depending on its word class (for a crosslinguistic analysis of iconicity by lexical classes, see Perlman et al., 2018). Therefore, we tested to what extent the degree of iconicity differed depending on the word classes of the corresponding word on the sign cards. As our data were not normally distributed according to the Shapiro–Wilk test (W = 0.92, *p* = 0.009), we used a nonparametric Kruskal-Wallis test.

In addition, we wanted to examine if the input provided by the educators was a potential confounding factor on children’s sign learning regarding iconicity. To this end, we analyzed video data and compared the degree of iconicity of the signs produced by the educators to the median degree of the 40 signs of the sign material. From the video recordings 6 months after implementation, 20 min per child were coded (children were visible on average over 90 percent of the time). Signs used by the educators were recorded each time they were used in interactions with the coded children. Since signs and gestures have areas of overlap (Kendon, 2008; Goldin-Meadow and Brentari, 2017), two hearing advanced signers categorized them following a systematic scheme based on Fricke (2007, 2012) (see Supplementary Material B). Twenty-three out of the total of 111 categorized signs were part of the 40 signs that had been rated for iconicity by hearing non-signers (see Appendix). As the Shapiro–Wilk test revealed no normal distribution of the median degree of iconicity of the 40 signs of the material (W = 0.92, *p* < 0.05) as well as of the 23 signs produced by the educators (W = 0.87, *p* < 0.05), a non-parametric Wilcoxon rank sum test was applied to compare their median degree of iconicity in order to check if educators used signs with a higher degree of iconicity more frequently than signs with a lower degree of iconicity.

## Results

**Figure 2:**
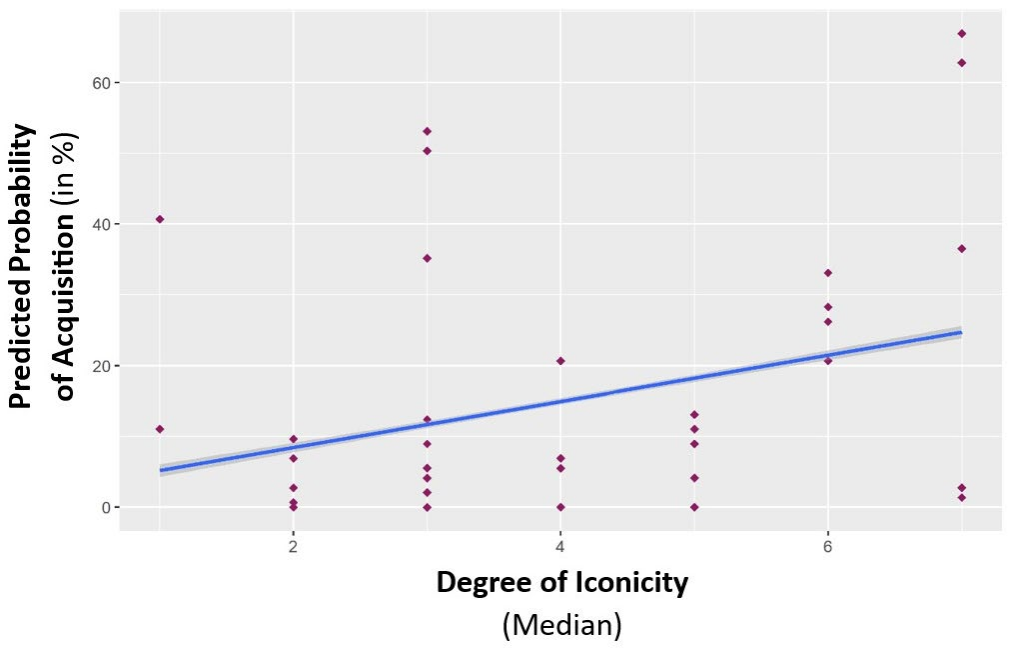
Percentage of signs acquired by all children in relation to the median degree of iconicity.

We observed a significant main effect of *language score* [χ(1) = 40.29, *p* < 0.001, see Table 3 for the full results of the GLMM], suggesting that whether a child learned a sign depended on their spoken language skills. In addition, we observed a significant main effect of *age* [χ(1) = 4.91, *p* = 0.027]. These effects were already found and analyzed in the previous study with these data by Schüler et al. (2021) revealing that children with better spoken language skills and older children were more likely to learn signs. There were effects of the *degree of iconicity* [χ (1) = 2.85, *p* = 0.092, see Figure 2] and of *early* *versus* *late acquisition of German language* [χ(1) = 3.41, *p* = 0.065], however, these were only trends.

**Table 3:** Results of the GLMM, with acquisition as the dependent variable.

Effect	Chisq	df	*p* value
*Degree of iconicity* (*z-scaled*)	*2.846*	*1*	*0.092*
Age (*z*-scaled)	**4.906**	**1**	**0.027**
Language score (*z*-scaled)	**40.287**	**1**	**<0.001**
*Early* *versus* *late acquisition of German language*	*3.413*	*1*	*0.065*
SEND	0.883	1	0.347
Degree of iconicity × Age	**8.607**	**1**	**0.003**
Degree of iconicity × Language score	1.727	1	0.189
Degree of iconicity × Early versus late acquisition of German language	0.069	1	0.792
*Degree of iconicity* × *SEND*	*2.816*	*1*	*0.093*

All continuous variables were *z*-transformed.

**Figure 3:**
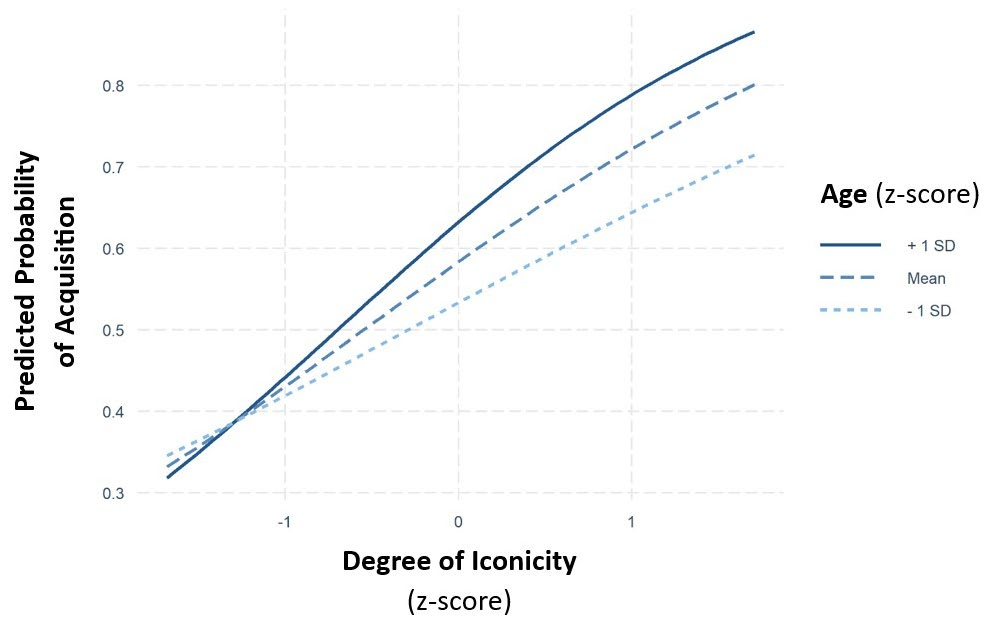
Predicted probability of acquisition in relation to the degree of iconicity (*z*-scaled), grouped by age (*z*-scaled).

**Figure 4:**
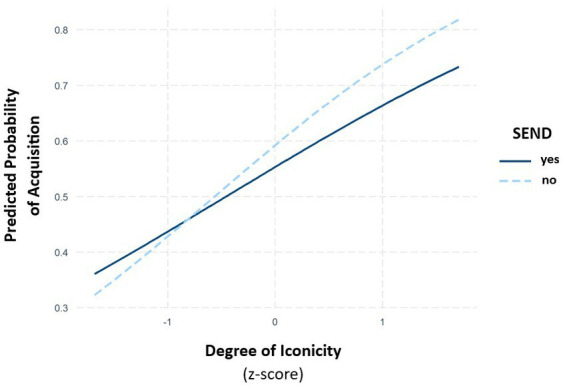
Predicted probability of acquisition in relation to the degree of iconicity (*z*-scaled), grouped by SEND.

There was a significant interaction between the *degree of iconicity* and *age* (χ = 8.61, *p* = 0.003). Consistent with our hypotheses, *post hoc* tests revealed that older children benefitted more from iconicity than younger ones (see Figure 3; Table 4 for EMMs and Table 5 for the *post hoc* tests). Moreover, there was a marginally significant two-way interaction between the *degree of iconicity* and *SEND*. Given that we had *a priori* hypotheses that children with and without SEND would differ in their gain from iconicity, we compared the slopes for degree of iconicity between the two groups. These comparisons revealed that children without SEND seem to benefit more from iconicity during their sign acquisition than children with SEND (β = 0.194, SE = 0.116, df = 579, *t* = 1.68, *p* = 0.093, see Table 6 for EMMs; see Figure 4). We found no interaction between the *degree of iconicity* and the *language score* of the children as well as between the *degree of iconicity* and *early versus late acquisition of German language*.

**Table 4:** EMMs calculating the trend of the variable *degree of iconicity* for three age groups.

Age	*Degree of iconicity* trend	SE	df	Lower.CL	Upper.CL
– 1 SD	0.389	0.401	5,787	−0.397	1.175
Mean	0.547	0.395	5,787	−0.228	1.321
+ 1 SD	0.704	0.396	5,787	−0.073	1.481

*Z*-scaled age was grouped into mean, one SD below mean and one SD above mean.

**Table 5:** Results of the *post hoc* tests assessing the effect of *age* (*z*-scaled) on the acquisition of signs with a higher *degree of iconicity* (*z*-scaled).

Contrast	Estimate	SE	df	*t*-ratio	*p* value
−1 SD vs. Mean	−0.158	0.054	5,787	−2.934	0.009
−1 SD vs. +1 SD	−0.315	0.107	5,787	−2.934	0.009
Mean vs. +1 SD	−0.158	0.054	5,787	−2.934	0.009

*Z*-scaled age was grouped as described in Table 4.

**Table 6:** Trends in the *degree of iconicity* for children without and with SEND.

SEND	*Degree of iconicity* trend	SE	df	Lower.CL	Upper.CL
No	0.644	0.395	5,787	−0.130	1.417
Yes	0.449	0.404	5,787	−0.342	1.241

The Kruskal-Wallis test revealed no significant differences between the degree of iconicity of different word classes [χ^2^(2) = 0.883, *p* = 0.830, *η*^2^ = −0.059].

The comparison of the degree of iconicity of the educator-produced signs and the signs of the training material revealed no significant differences (W = 494, *p* = 0.624). This suggests that the educators did not use significantly more signs with a higher degree of iconicity from the training material.

## Discussion

In the current study, we investigated the extent to which the iconicity of signs influences children’s vocabulary learning in the natural setting of inclusive day care centers. We found that iconicity affected the acquisition of signs, but the strength of this effect depended on the participants’ age. Consistent with our prediction, older children learned significantly more signs with a higher degree of iconicity than younger children did. Contrary to previous assumptions, children with SEND in our sample did not benefit more from iconicity than children without SEND.

Our finding that children are more likely to learn signs with a higher degree of iconicity is in line with previous findings of non-signing children’s vocabulary in spoken languages, providing further evidence for the notion that children are more likely to learn words with a higher degree of iconicity (Perry et al., 2015, 2017; Perlman et al., 2017; Sidhu et al., 2021). This refers to sound symbolism (Maurer et al., 2006; Imai et al., 2008; Peña et al., 2011; Ozturk et al., 2013; Massaro and Perlman, 2017; Tzeng et al., 2017) as well as to onomatopoeia (Laing, 2019; Motamedi et al., 2021). Furthermore, these results are consistent with most sign vocabulary analyses of deaf children at a comparable age, where iconicity has also been shown to increase the likelihood that signs are part of active vocabulary (Thompson et al., 2012; Caselli and Pyers, 2017, 2020). Thus, it seems that non-signing children are sensitive to iconicity and use it not only in their first language and its modality (e.g., Perry et al., 2015, 2017; Massaro and Perlman, 2017; Perlman et al., 2017; for a review see Nielsen and Dingemanse, 2021) but also in the learning of signs as indicated by some older studies (Brown, 1977 and Page, 1982 in Luftig, 1982). Similar results have also been observed in experimental studies with non-signing children investigating the learning of iconic gestures (Marentette and Nicoladis, 2011; Magid and Pyers, 2017) and the recognition of iconic signs (Tolar et al., 2008). Studies in sign-naïve adults reveal that the implicit knowledge of iconic gestures might facilitate the production of depicting signs (Kurz et al., 2023) and scaffold the production and recognition of iconic signs (e.g., Ortega et al., 2020, 2022; for an overview, see Chen Pichler and Koulidobrova, 2016). However, after a short practice period, iconic signs with a large or a small overlap with iconic gestures seemed to be learned with similar success (Ortega et al., 2020). Thus, the extent to which this effect might play a role in our long-term study is not clear. Moreover, the effect we found in our study was statistically marginal for the overall group and differed for the various subgroups.

In particular, older children learned more signs with a higher degree of iconicity, whereas younger children did not seem to benefit from iconicity to the same degree. This finding is in line with Thompson et al. (2012), who investigated the role of iconicity in deaf children’s acquisition of sign language, with Magid and Pyers (2017) and Marentette and Nicoladis (2011) for the learning of iconic and arbitrary gestures by non-signing children, and Tolar et al. (2008) for the recognition of iconic signs by non-signing children (for similar results with iconic gestures, see Magid and Pyers, 2017). The greater benefit of iconicity for older children has been explained by a developing sensitivity to iconicity with increasing age (e.g., Tolar et al., 2008; Marentette and Nicoladis, 2011; Thompson et al., 2012). Most researchers agree that the cognitive abilities for recognizing and exploiting iconicity in signs become more stable around 3 years of age and continue to increase with age (e.g., Tolar et al., 2008; Thompson et al., 2012; Caselli and Pyers, 2017). It has often been hypothesized that the maturation of various cognitive domains such as spatial cognition, analogical reasoning abilities, or metalinguistic abstraction abilities are important prerequisites for this, but especially experiences seem to play a crucial role (Tolar et al., 2008; Perniss and Vigliocco, 2014; Magid and Pyers, 2017; Karadöller et al., 2022). This also seems to apply to the recognition and exploitation of sound symbolism (e.g., Tzeng et al., 2017). Despite innate mechanisms, also learned mechanisms are required for exploiting iconicity (Fort et al., 2018).

Consistent with Tolar et al. (2008), our data suggest that chronological age seems to be more crucial than language age in a spoken language for the extent to which children capitalize on iconicity in their sign learning. By contrast, when analyzing sign learning independently of iconicity, language age in a spoken language and not chronological age seemed to be the determining factor (Schüler et al., 2021). Accordingly, Joyce et al. (2023) have shown that language-based skills are crucial for sign learning in adults irrespective of cognitive (dis)abilities. This suggests that language acquisition *per se* does not require cognitive abilities that are necessary to recognize iconicity. But as soon as these cognitive abilities are developed, iconicity can be exploited and promote sign vocabulary learning. That deaf signing preschool children seem to capitalize on iconicity at a younger age than sign-naïve preschool children when recognizing and learning gestures (Magid and Pyers, 2017) suggests that experiences with a language rich in iconicity may enhance the process of exploiting iconicity. As these age effects are found for signs (Thompson et al., 2012) and gestures (Marentette and Nicoladis, 2011; Magid and Pyers, 2017) an analysis of gestures in inclusive day care groups might have produced similar results. The findings of Marshall and Hobsbaum’s (2015) study of word learning in bilingual children whose teachers used speech-accompanying signs or more spontaneous gestures also suggest that signs and gestures may lead to similar effects. Moreover, recent research on hearing and deaf children emphasizes the commonalities of gestures and signs (Capirci et al., 2023).

Previously, it has been suggested that iconicity might be an important factor in why some children with SEND learn signs more easily than spoken words, revealing that children with SEND could have an especially significant gain from iconicity for sign acquisition (Konstantareas et al., 1978; Bonvillian et al., 1981; Lloyd et al., 1985). However, in our study with a heterogenous sample, children with SEND did not seem to benefit more from iconicity than children without SEND. Children with SEND in our sample even seemed to exploit iconicity less or later than children without SEND, but the effect we observed was only a trend. To benefit from iconicity, researchers suggest that not only experiences in the world must be gained, but also the cognitive abilities need to be developed that help to abstract from these experiences and to identify salient features of signs or gestures in order to link referent and linguistic form (Tolar et al., 2008; Perniss and Vigliocco, 2014; Magid and Pyers, 2017). Although it has not yet been conclusively clarified to what extent the development of which cognitive domains and to what extent which experiences play a role in this process, it can be assumed that both cognitive abilities and experiences proceed with increasing age (Magid and Pyers, 2017).

It can be hypothesized that in some children with SEND some cognitive areas relevant for exploiting iconicity may develop slower compared to children without SEND. Indeed, most children with SEND in previous studies (Konstantareas et al., 1978; Bonvillian et al., 1981; Lloyd et al., 1985; Bello et al., 2004; Stefanini et al., 2007; Dargue et al., 2021) were older than the children with SEND in our study. Based on this, it could be assumed that these older children’s abilities to use iconicity were more developed than in our young preschool sample. However, it could also be possible that children with certain diagnoses, which are not present or are too few in numbers for analyses in our sample, might benefit more from iconicity than children without SEND. It should be noted that in our study we considered all children with SEND as one group, regardless of their individual needs. To further analyze how the effects of iconicity possibly differ between children with different special educational needs and/or disabilities, larger sample sizes with homogenous groups of children concerning their diagnoses are needed.

Additionally, in contrast to experimental studies, studies in natural settings have little control over the input that adults provide to children (for spoken language, see Sidhu et al., 2021; for sign language, see Thompson et al., 2012; Caselli and Pyers, 2017; for sign supported communication see Marshall and Hobsbaum, 2015). Studies on hearing adult sign language learners show that they are very sensitive to iconicity, which speeds lexical access (Mott et al., 2020) and that they are more likely to use iconic signs (Lieberth and Gamble, 1991; Baus et al., 2013). By contrast, in child-directed signing, Gappmayr et al. (2022) found iconic signs not to be overrepresented in the signing of deaf and hearing parents toward their deaf children. To assess whether the educators’ input regarding the frequency of iconic signs was a biasing factor in our sample, we additionally analyzed video data 6 months after sign implementation in the groups. Comparing the average degree of iconicity of the signs produced by the educators with the average degree of iconicity of the signs in this study, the median value did not differ significantly. Although this observation was made on a small dataset and only on a subset of educators, it seems that the children’s sign learning behavior observed in our study regarding iconicity was probably not systematically biased by sign frequency in the educators´ input.

Other factors that may affect sign acquisition besides the degree of iconicity should also be investigated with a larger number of items in more detail, as found in deaf children for frequency, neighborhood density (Caselli and Pyers, 2017), concreteness, babiness (Caselli and Pyers, 2020), and, with diverging results, type of iconicity (Ortega et al., 2017; Caselli and Pyers, 2020).

## Conclusion

Our results suggest that iconicity affects the learning of signs in inclusive day care settings. This especially applies to older children. The extent to which a group of preschool children with certain special educational needs and/or disabilities may benefit from iconicity, depending on their skills, needs to be explored in larger studies.

## Data availability statement

The original contributions presented in the study are publicly available. This data can be found here: https://doi.org/10.25592/uhhfdm.12540.

## Ethics statement

The study was performed in accordance with the ethical standards as laid down in the 1964 Declaration of Helsinki and its later amendments of comparable ethical standards, and the project was approved by the Federal Ministry of Education and Research. The informed consent form was reviewed by the Leibniz Institute for Human Development and Educational Information. Written informed consent was obtained from the minors' legal guardian/next of kin for the publication of any potentially identifiable images or data included in this article.

## Author contributions

MG-K and BH-F contributed to the conceptualization and design of the study. MG-K conducted the investigation and wrote the original draft. MG-K and A-LS performed the formal analysis. MG-K, A-LS, and BH-F were involved in writing, reviewing, and editing of the manuscript. BH-F supervised and provided funding for the investigation. All authors contributed to the article and approved the submitted version.

## Funding

This study was funded by the German Federal Ministry of Education and Research (BMBF FKZ: 01NV1706), awarded to BH-F and the Open Access Publication Fund of Universität Hamburg, awarded to MG-K.

## Conflict of interest

The authors declare that the research was conducted in the absence of any commercial or financial relationships that could be construed as a potential conflict of interest.

## Publisher’s note

All claims expressed in this article are solely those of the authors and do not necessarily represent those of their affiliated organizations, or those of the publisher, the editors and the reviewers. Any product that may be evaluated in this article, or claim that may be made by its manufacturer, is not guaranteed or endorsed by the publisher.

## Footnotes

^1^Based on Schüler and Hänel-Faulhaber (2023), we expected that the age of onset could have a greater impact on sign acquisition and the role of iconicity in this process than bilingualism *per se.*
