# Supplementary material for: Sign learning of hearing children in inclusive day care centers—does iconicity matter?
Source: Front Psychol. 2023 Aug 16;14:1196114. doi: 10.3389/fpsyg.2023.1196114 (PMC10467423; doi:10.3389/fpsyg.2023.1196114)
Supplement: Supplementary file 1 [file Data_Sheet_1.pdf]

SUPPLEMENTARY MATERIAL

Supplementary Material A: A translation of the educator questionnaire is provided here. The questionnaire contains questions regarding demographic information and three sections assessing vocabulary (Part 1), grammar (Part 2), and an additional section concerning communication that is not analyzed for the purpose of this paper and therefore, not presented here.

Questionnaire on child language development

(for educational professionals)

General information

Child's name: ____

Gender: ☐ girl ☐ boy

Date of birth: ____ /____ /________

Today's date: ____ /____ /________

Name of kindergarten:

Name of kindergarten group:

Does the child receive any special educational support/therapy? ☐ yes ☐ no

If YES, please provide details in keywords:

Has the child been diagnosed with permanent hearing loss? ☐ yes ☐ no

If YES, please provide details below:

	right ear:	*left ear*:
Degree of hearing loss in dB		
Supply with hearing aid (HA) or cochlear implant (CI)	HA ☐ CI ☐<br>since_________	HA ☐ CI ☐<br>since_________

Additional information:

Does the child have physical disabilities, chronic diseases or other permanent impairments? ☐ yes ☐ no

If YES, please provide details in keywords:

Does the child grow up multilingual? ☐ yes ☐ no

If YES, please indicate all languages here:

If YES, since when has the child been learning German? *(Example: since 05/2014):*

If the child grows up multilingual, please note that the information in the following questionnaire only refers to what the child says in **German.** In addition, **signs of German Sign Language** are recorded.

Important! The assessment should be carried out by a person who has known the child **very well for at least 3 months**. The first and second assessment should be done by the **same person**.

Part 1 - Vocabulary

Subsequently, you will find a list of words that young children typically have in their vocabulary.

There are a lot of words listed here as this is a collection of words used by different children and also by children who may be a bit older. So don't worry if the child only speaks a few of these words at this point. Go through the list and tick the words that the child **speaks and/or signs**.
However, only words/signs should be ticked that the child has used more than once and not those that it only repeats or understands. Please also tick words that the child pronounces slightly differently (e.g., “taufen” (German word for "baptize") instead of “kaufen” (German word for "buy") or “daußen" (German word for “outside” but with omitted “r”) instead of “draußen” (German word for “outside”). If the child uses something similar (e.g., "cup" for "glass"), please write that next to the respective entry.

If the child uses both the word and the sign, please tick both.

	speaks	signs		speaks	signs		speaks	signs		speaks
birthday	☐	☐	work	☐	☐	small	☐	☐	to run	☐
girl	☐	☐	to water	☐	☐	to cook	☐	☐	with	☐
to need	☐	☐	to say	☐	☐	outdoors	☐	☐	glass	☐
head	☐	☐	tired	☐	☐	sand	☐	☐	bucket	☐
to eat	☐	☐	ready	☐	☐	later	☐	☐	to like	☐
throat	☐	☐	to laugh	☐	☐	music	☐	☐	clean	☐
towel	☐	☐	to find	☐	☐	lamp	☐	☐	have to	☐
spicy	☐	☐	finger	☐	☐	today	☐	☐	quiet	☐
give a gift	☐	☐	wet	☐	☐	meat	☐	☐	to listen	☐
light	☐	☐	new	☐	☐	umbrella	☐	☐	to fly	☐
to clean	☐	☐	I	☐	☐	more	☐	☐	to play	☐
to taste	☐	☐	nice	☐	☐	ear	☐	☐	now	☐
breakfast	☐	☐	to buy	☐	☐	to lie (down)	☐	☐	paper	☐
dirty	☐	☐	sun	☐	☐	carpet	☐	☐	warm	☐
to cut	☐	☐	to live	☐	☐	fast	☐	☐	to jump	☐
animal	☐	☐	to wait	☐	☐	cloud	☐	☐	to stand	☐
chocolate	☐	☐	table	☐	☐	to wash	☐	☐	tooth	☐
to tidy up	☐	☐	once again	☐	☐	to want	☐	☐	alone	☐
cabinet	☐	☐	stone	☐	☐	tomato	☐	☐	water	☐
toe	☐	☐	shoe	☐	☐	pen	☐	☐	stairs	☐
away	☐	☐	room	☐	☐	heavy	☐	☐	street	☐
full	☐	☐	soft	☐	☐	tongue	☐	☐	to search	☐
to swim	☐	☐	to read aloud	☐	☐	meadow	☐	☐	to see	☐
together	☐	☐	soup	☐	☐					

Part 2 - Grammar

1. Does the child already use word combinations/sentences of two or more words, such as "mommy book", "playing baby" or "Is that going in there?"? ☐ yes ☐ no

If you ticked YES, please answer all subsequent questions. If you ticked NO, please continue to 'Part 3 – communication'.

In the following, always tick the option that **most closely** corresponds to what the child would say.

2. ☐ There cat.
   ☐ There's a cat.
3. ☐ Mommy shopping.
   ☐ Mommy is shopping.
4. ☐ This mine!
   ☐ This is mine!
5. ☐ Mommy cook.
   ☐ Mommy is cooking.
6. ☐ many car
   ☐ many cars
7. ☐ many flower
   ☐ many flowers
8. Does the child use the sentence connector **and**? (e.g., "I'll get the book **and** then you read it.") ☐ yes ☐ no
9. Does the child use the words my (grammatical gender male)/my (grammatical gender female) correctly? (e.g., **my** room, **my** doll, **my** toys) ☐ yes ☐ no

10 If the child doesn't want an apple, they're more likely to say:
☐ "Don't eat apple!"
☐ "I don't want to eat an apple!"

Does the child use the question word:

11. **How?** – e.g., "How does this game work?" ☐ yes ☐ no
12. **What?** – e.g., "What have you got there?" ☐ yes ☐ no
13. **Where?** - e.g., "Where's my ball?" ☐ yes ☐ no
14 **To where?** – e.g., "Where is Dad going?" ☐ yes ☐ no

15 Does the child retell short stories/fairy tales (using pictures)? ☐ yes ☐ no

[...]

Supplementary Material B: The classification scheme for defining signs and gestures in our video data is provided here. It was developed according to Fricke (2007, 2012). The signs or gestures were assigned to the respective categories by two hearing advanced signers. Inter-Rater-Agreement was calculated using Cohen´s Kappa (Cohen, 1960) in the R package *DescTools* (Signorell, 2023). Coders showed almost perfect agreement (κ = 0.83, 95% CI[0.77, 0.88]).

Classification of Signs and Gestures in our Data

Abbreviations: DGS = Deutsche Gebärdensprache (German Sign Language)

**Category**		**Definition**
1	Gesture	Criteria:<br>Fulfillment of 1.1. and, in case of only one parameter deviation from lexicalized signs from DGS, one of the criteria of 1.2
		1.1. The execution differs in at least one parameter from a DGS sign that semantically matches the word or context.
		1.2. If deviating in exactly one parameter, one of the following criteria must apply:<br><br>1.2.1. Typical use for referring iconic or deictic gestures (imitation of actions, representation of forms, indication of size, localization in space)<br>1.2.2. If regarded as referring arbitrary gesture (which is rarely used following Fricke, 2007, p. 218), there must be evidence for their widespread use.<br>1.2.3. Produced in a typical manner for non-referring gestures (e.g., repetitive strokes to emphasize or change discourse levels, ideographic, performative)<br><br>(According to the classification scheme of speech-accompanying gestures by Fricke 2007, p. 222)
2	Sign/Gesture	Criteria:<br>2.1. and 2.2. must be met:
		2.1. A lexicalized DGS sign is produced that deviates in maximum one parameter from the linguistically correct form, but at the same time it corresponds to a common gesture (category 1 of this scheme). Accordingly, at least one of the criteria of 1.2. must apply.<br>2.2. This does not occur within an interaction in which at least one other sign (category 3) is produced by the same person.

3	Sign	Criteria:<br>One of the following criteria (3.1 to 3.4) must be met:
		3.1. A correctly produced lexicalized DGS sign is used, which does not correspond to any common gesture.
		3.2. A correctly produced lexicalized DGS sign is used that also corresponds to a common gesture according to 1.2, but occurs within an interaction with at least one correctly produced lexicalized DGS sign according to 3.1.
		3.3. A lexicalized sign may not be performed correctly in maximum one parameter, but a gesture is unlikely because none of the criteria of 1.2 applies.
		3.4. A lexicalized sign is produced incorrectly in more than one parameter but is clearly labeled as a sign in context (incorrect production).
4	Number(word)	Numbers are assigned to this category because they are often produced (additionally) in the visual-spatial modality, both as signs and gestures, and to assign them to categories 1 to 3 is particularly difficult.<br><br>Exception: If a in the visual-spatial modality produced number occurs within interactions with signs from category 3, it is also assigned to category 3.
5	Pronoun	Pronouns are assigned to this category because they are produced not only as signs, but also as gestures often for emphasis additionally in the visual-spatial modality and to assign them to categories 1 to 3 is particularly difficult.<br><br>Exception: If a in the visual-spatial modality produced pronoun occurs within interactions with signs from category 3, it is also assigned to category 3.
6	Sign in metalinguistic communication	A sign, which does not need to be clearly distinguishable from a gesture, is used or specifically practiced in metalinguistic communication (e.g., looking at sign cards, asking for signs).<br><br>Hierarchy for assignment: at the top (e.g. if a sign, a number word or a pronoun is used in metalinguistic communication, always 6, not 3, 4 or 5).
7	Sign in song	A sign, which does not need to be clearly distinguishable from a gesture, is used when singing a song.<br><br>Hierarchy for assignment: at the top (e.g. if a sign, a number word or a pronoun is used in a song, always 7, not 3, 4 or 5).

References:

Cohen, J. (1960). A Coefficient of Agreement for Nominal Scales. Educational and Psychological Measurement, 20(1), 37–46. https://doi.org/10.1177/001316446002000104

Fricke, E. (2007). *Origo, Geste und Raum—Lokaldeixis im Deutschen*. De Gruyter.

Fricke, E. (2012). *Grammatik multimodal. Wie Wörter und Gesten zusammenwirken*. Walter de Gruyter.

Signorell, A. (2023). DescTools: Tools for Descriptive Statistics. R package version 0.99.4. https://CRAN.R-project.org/package=DescTools

Suchodoletz, W. v., Kademann, S., & Tippelt, S. (2011). *Sprachbeurteilung durch Eltern – Kurztest für die U7a (SBE-3-KT). (“Language assessment by parents—Short test for child screening U7a”)*. https://www.kjp.med.uni-muenchen.de/download/SBE-3-KT_Handbuch.pdf
