# Supplementary material for: Sign learning of hearing children in inclusive day care centers—does iconicity matter?
Source: Front Psychol. 2023 Aug 16;14:1196114. doi: 10.3389/fpsyg.2023.1196114 (PMC10467423; doi:10.3389/fpsyg.2023.1196114)
Supplement: Supplementary file 2 [file Data_Sheet_2.pdf]

Appendix

The rating procedure for the degree of iconicity of the analyzed signs and its results are provided here.

Iconicity Rating Procedure

The 40 signs that were part of the questionnaire (see Supplementary Material A) and of the 232 signs of the material for the educators and the children (for an example, see Figure 1) were selected. These 40 signs consisted of 11 nouns, 9 verbs, 11 adjectives, and 9 words of other types. For determining the degree of iconicity, 180 hearing students were asked to rate the signs via an online questionnaire. At the beginning, normal or corrected to normal vision and no prior sign language experience had to be confirmed, otherwise the questionnaire was stopped. To include only participants without knowledge of sign language is consistent with Caselli et al. (2017) for ASL-LEX to avoid biases due to prior knowledge (see Vinson et al., 2008). 147 students (mean age: 28.0 years, range: 19 – 58 years; 109 female, 36 male, 2 diverse) fulfilled the requirements and completed the questionnaire.

Participants were asked to rate the iconicity of the signs on a 7-point Likert scale (1 = not iconic at all; 7 = highly iconic, see Caselli et al., 2017; Vinson et al., 2008). If the signs were depicting the shape or action of the referent or meaning very well, participants were instructed to choose high values corresponding to a high degree of iconicity. By contrast, they should assign low values if they did not see any connection between aspects of the signs and their meaning. Participants were instructed to answer as quickly as possible. After three practice trials that were not included in the analysis, every sign was presented via video with the German translation above. Each sign was produced by a native signer. This was followed by the 7-point Likert scale, which remained on the screen for five seconds. If participants missed answering within this time span, the next item was presented to them. Missing values of this kind (2.94%) were excluded from the analysis.

Rated and Analyzed Signs

**SIGN *(translation)***	**Degree of Iconicity** Mean/Median	**Degree of Iconicity** Mean/Median (Trettenbrein et al., 2021)	**Acquired by**
ICH *(I/me)*	6.81 / 7	-	62.8%
LEISE *(quiet/silent)*	6.58 / 7	-	36.6%
ESSEN *(to eat)*	6.38 / 7	6.23/7	66.9%
KOPF *(head)*	6.28 / 7	-	2.6%
SEHEN *(see)*	6.28 / 7	-	1.4%
KLEIN *(small)*	6.04 / 6	-	28.3%
SONNE *(sun)*	5.70 / 6	-	33.1%
WARTEN *(wait)*	5.57 / 6	other sign variant	20.7%
MUSIK *(music)*	5.48 / 6	5.97/6.5	26.2%
ZUSAMMEN *(together)*	5.28 / 5	-	13.1%
DRAUSSEN *(outside)*	5.14 / 5	-	11.0%
SCHWER *(heavy)*	5.03 / 5	-	0.0%
JETZT *(now)*	4.96 / 5	-	9.0%
MÜSSEN *(must/need to)*	4.82 / 5	-	0.0%
SUPPE *(soup)*	4.76 / 5	-	0.0%
HEUTE *(today)*	4.46 / 5	-	4.14%
TISCH *(table)*	4.28 / 4	4.37 / 4.5	6.9%
VOLL *(full)*	3.94 / 4	-	0.0%
NOCHMAL *(again)*	3.89 / 4	-	19.5%
GLAS *(glass)*	3.64 / 4	-	5.5%
SPÄTER *(later)*	3.50 / 3	-	9.0%
AUFRÄUMEN *(to tidy up)*	3.31 / 3	2.4 / 2	50.3%
SAUBER *(clean)*	3.26 / 3	-	0.0%
ARBEITEN *(to work)*	3.24 / 3	-	35.2%
FERTIG *(ready)*	3.23 / 3	-	53.1%
MIT *(with)*	3.06 / 3	-	0.0%
MÜDE *(tired)*	3.03 / 3	-	12.4%
MÖGEN *(to like)*	2.99 / 3	-	5.5%
SCHNELL *(fast/quick)*	2.90 / 3	-	2.1%
SCHMUTZIG *(dirty)*	2.88 / 3	-	0.0%
ALLEIN *(alone)*	2.71 / 3	-	4.1%
SCHUHE *(shoes)*	2.64 / 2	-	0.7%
WASSER *(water)*	2.62 / 2	-	2.8%
MEHR *(more)*	2.57 / 2	other sign variant	6.9%
GEBURTSTAG *(birthday)*	2.50 / 2	-	9.7%
WOLLEN *(to want to)*	2.35 / 2	-	2.8%
NASS *(wet)*	2.35 / 2	-	0.0%
FLEISCH *(meat)*	1.88 / 2	-	0.0%
MÄDCHEN *(girl)*	1.81 / 1	1.27 / 1	11.0%
SPIELEN *(play)*	1.80 / 1	-	40.7%

References:

Caselli, N. K., Sehyr, Z. S., Cohen-Goldberg, A. M., & Emmorey, K. (2017). ASL-LEX: A lexical database of American Sign Language. Behavior Research Methods, 49(2), 784–801. https://doi.org/10.3758/s13428-016-0742-0

Trettenbrein, P. C., Pendzich, N.-K., Cramer, J.-M., Steinbach, M., & Zaccarella, E. (2021). Psycholinguistic norms for more than 300 lexical signs in German Sign Language (DGS). *Behavior Research Methods*, *53*(5), 1817–1832. https://doi.org/10.3758/s13428-020-01524-y

Vinson, D. P., Cormier, K., Denmark, T., Schembri, A., & Vigliocco, G. (2008). The British Sign Language (BSL) norms for age of acquisition, familiarity, and iconicity. Behavior Research Methods, 40(4), 1079–1087. https://doi.org/10.3758/BRM.40.4.1079
